# Pancreatic Perivascular Epithelioid Cell Tumor: A Case Report and Literature Review

**DOI:** 10.70352/scrj.cr.26-0589

**Published:** 2026-10-01

**Authors:** Chie Kitami, Yasuyuki Kawachi, Toshihiko Ikarashi, Atsushi Nishimura, Tetsuya Nakano, Shigeto Makino, Mikako Kawahara

**Affiliations:** 1 Department of Surgery, Nagaoka Chuo General Hospital, Nagaoka, Niigata, Japan; 2 Department of Pathology, Nagaoka Chuo General Hospital, Nagaoka, Niigata, Japan

**Keywords:** pancreatic PEComa, perivascular epithelioid cell tumor, Folpe classification, endoscopic US-guided fine-needle biopsy

## Abstract

**INTRODUCTION:**

Perivascular epithelioid cell tumors (PEComas) are rare mesenchymal neoplasms, and primary pancreatic PEComas are exceptionally uncommon. Owing to their rarity, the prognostic significance of their pathological features remains unclear. We report a resected case of pancreatic PEComa and review the literature to further characterize its clinicopathological features.

**CASE PRESENTATION:**

A 72-year-old woman presented with a gradually enlarging, 63-mm hypervascular tumor in the pancreatic body. Contrast-enhanced CT and MRI demonstrated a well-circumscribed hypervascular mass with central hypoenhancement, suggestive of coagulative necrosis. Endoscopic US-guided tissue acquisition suggested acinar cell carcinoma because of weak trypsin positivity, and distal pancreatectomy with regional lymph node dissection was performed. Histopathological examination of the resected specimen revealed epithelioid and spindle cells that were positive for HMB45 and Melan-A and weakly positive for α-smooth muscle actin, leading to a diagnosis of pancreatic PEComa. The patient remained disease-free at 18 months after surgery. We also reviewed 26 previously reported cases of pancreatic PEComa and re-evaluated those with sufficient pathological information according to the Folpe criteria. Metastasis developed in 1 tumor classified as malignant and 1 classified as having uncertain malignant potential. Coagulative necrosis and increased mitotic activity appeared to be more frequent in the 2 metastatic cases; however, the small number of events precluded conclusions regarding their prognostic significance or the predictive performance of the Folpe classification.

**CONCLUSIONS:**

Pancreatic PEComa should be considered in the differential diagnosis of hypervascular pancreatic tumors with atypical immunophenotypic findings. Additional immunohistochemical staining for melanocytic markers may facilitate an accurate preoperative diagnosis. The prognostic significance of individual pathological features and the applicability of the Folpe classification to pancreatic PEComa remain uncertain and require evaluation in additional well-documented cases with long-term follow-up.

## Abbreviations

α-SMA
: α-smooth muscle actin
EUS
: endoscopic US
EUS-TA
: endoscopic US-guided tissue acquisition
HMB45
: human melanoma black 45
PEC
: perivascular epithelioid cell
PEComa
: perivascular epithelioid cell tumor
UMP
: uncertain malignant potential

## INTRODUCTION

PEComas are rare mesenchymal neoplasms composed of distinctive PECs with multilineage differentiation potential.^1)^ Although PEComas have been reported in various organs, primary pancreatic PEComas are exceedingly rare. While most reported pancreatic PEComas have followed a favorable clinical course, metastatic and recurrent cases have also been described.^2,3)^ Owing to their rarity, the clinicopathological characteristics and prognostic factors of pancreatic PEComas remain poorly understood. Furthermore, the number of reported pancreatic PEComas varies among previous reviews, probably because of differences in case selection, possible duplication of cases, and variability in the pathological information available in the original reports. We herein report a resected case of pancreatic PEComa and review previously reported cases to further characterize its clinicopathological features.

## Case presentation

A 72-year-old woman with a history of malignant lymphoma had achieved complete remission after treatment with rituximab plus bendamustine. During routine follow-up CT performed at our hospital, a previously identified pancreatic body mass was found to have gradually increased in size, prompting a referral to our department for surgical evaluation.

Laboratory tests revealed a mildly elevated CA19-9 level of 49.3 U/mL, whereas all other tumor marker levels were within the normal range. Contrast-enhanced CT revealed a well-circumscribed, 63-mm mass in the pancreatic body (**Fig. 1**). The peripheral portion of the tumor showed marked enhancement during the pancreatic parenchymal phase, whereas the central portion showed relatively weak enhancement. The enhancement decreased on delayed-phase images, demonstrating a washout pattern. No dilatation of the main pancreatic duct or major vascular invasion was observed. MRI demonstrated that the lesion exhibited iso- to slightly high signal intensity on T2-weighted, fat-suppressed images (**Fig. 2A**). Dynamic contrast-enhanced MRI demonstrated peripheral enhancement in the early phase with delayed washout (**Fig. 2B**, **2C**). EUS revealed a well-defined, iso- to hyperechoic solid mass in the pancreatic body (**Fig. 3**). The lesion was predominantly homogeneous with focal heterogeneity. No obvious cystic components or calcifications were identified. Color Doppler imaging demonstrated abundant intratumoral vascular flow. EUS-TA revealed tumor cells with round-to-oval nuclei and pale eosinophilic to finely granular cytoplasm, without a characteristic architectural pattern. Immunohistochemical staining showed weak positivity for trypsin and negativity for keratin AE1/AE3, chromogranin A, synaptophysin, CD56, β-catenin, c-kit, CAM5.2, CK7, CK20, and S-100. Additional acinar markers, including chymotrypsin and BCL10, were not evaluated in the preoperative biopsy specimen. The Ki-67 labeling index was approximately 10%. Although the weak trypsin positivity was atypical for acinar cell carcinoma, acinar cell carcinoma was suspected because the examined neuroendocrine and epithelial markers were negative. At laparotomy, an approximately 6 cm, elastic, soft mass was identified in the pancreatic body. Distal pancreatectomy with regional lymph node dissection was then performed according to the standard procedure for pancreatic cancer. Macroscopically, the cut surface revealed a well-circumscribed, yellowish-white solid tumor with a relatively well-defined margin (**Fig. 4**). Histopathological examination revealed spindle-shaped and epithelioid cells with clear to eosinophilic cytoplasm arranged around blood vessels (**Fig. 5**). Focal coagulative necrosis was also observed. Immunohistochemical examination showed that the tumor cells were negative for keratin AE1/AE3, chromogranin A, synaptophysin, and CD56, thereby excluding pancreatic neuroendocrine neoplasms. In contrast, the tumor cells were positive for HMB45 and Melan-A, weakly positive for α-SMA, and negative for S-100 (**Fig. 6**). The Ki-67 labeling index was 5%. No mitotic figures were identified (0/50 high-power fields), no vascular invasion was identified, and focal coagulative necrosis was present. Based on these histopathological and immunohistochemical findings, the tumor was diagnosed as a pancreatic PEComa. The postoperative course was uneventful. The patient remained alive without recurrence at 18 months after surgery.

**Fig. 1:**
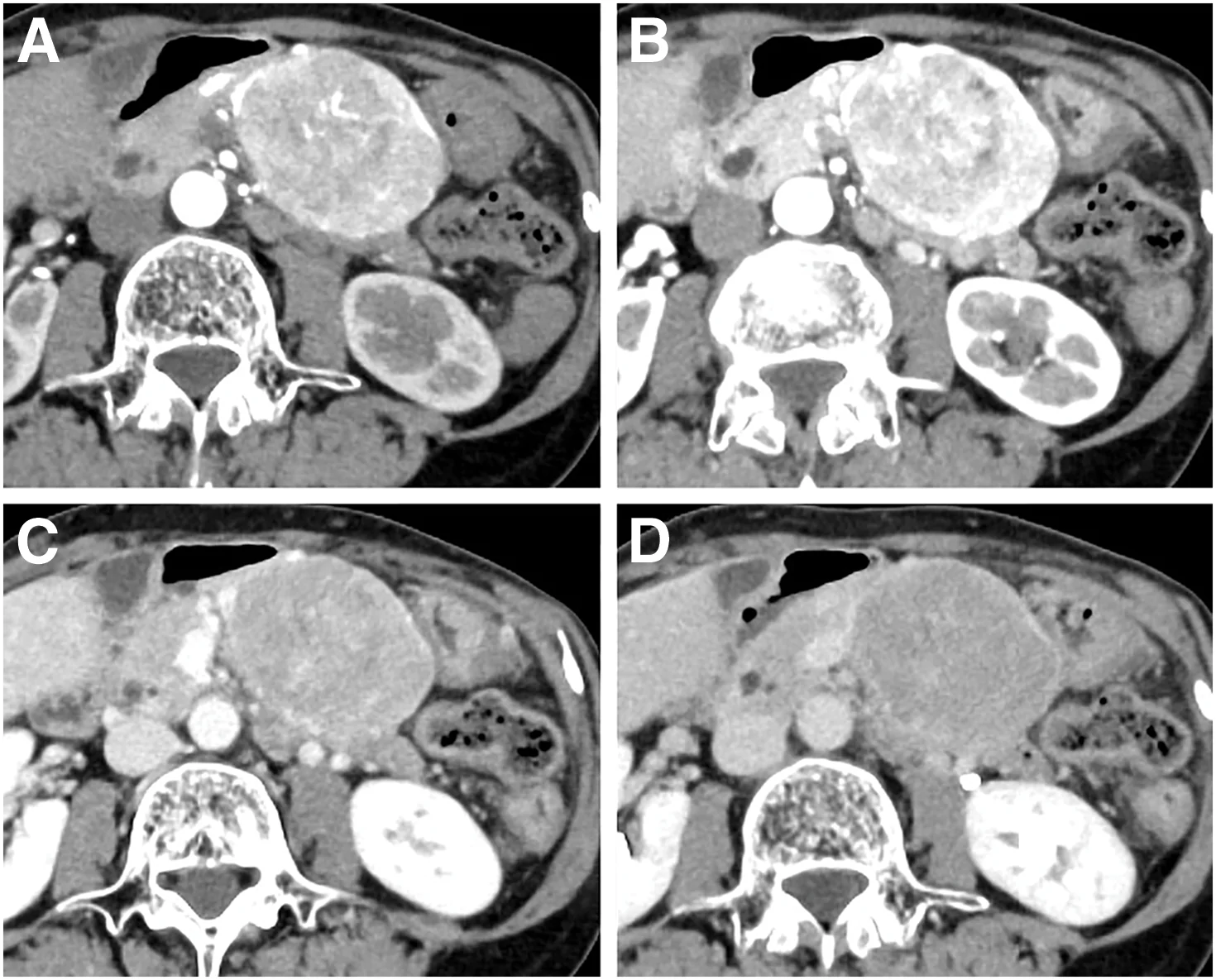
Contrast-enhanced CT findings. (**A**) Early arterial phase, (**B**) pancreatic parenchymal phase, (**C**) portal venous phase, and (**D**) delayed phase. A well-circumscribed hypervascular tumor was observed in the pancreatic body.

**Fig. 2:**
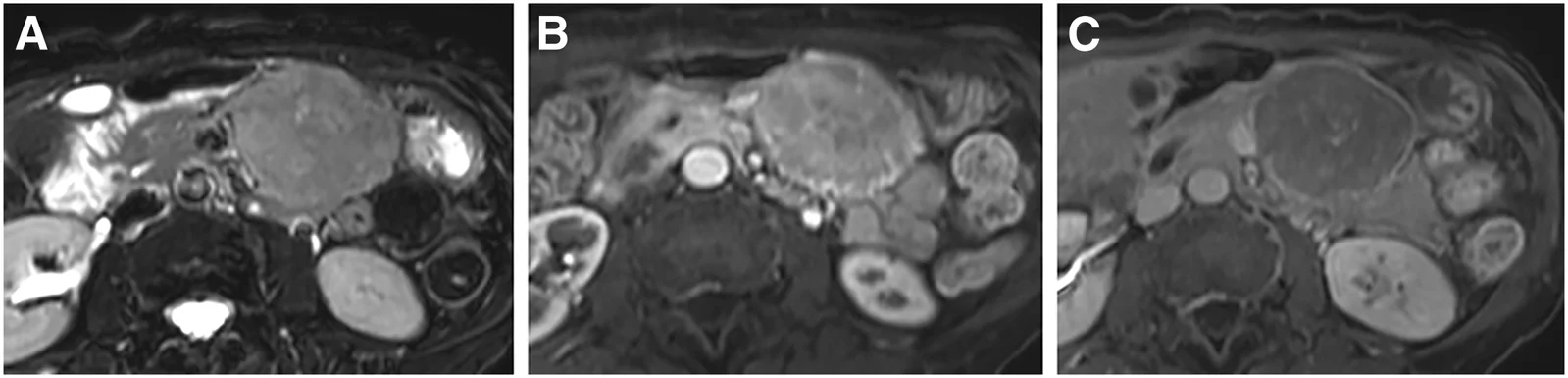
MRI findings. (**A**) T2-weighted, fat-suppressed image demonstrating a well-circumscribed mass with iso- to slightly high signal intensity. (**B**) Dynamic contrast-enhanced MRI demonstrating peripheral enhancement in the early phase. (**C**) Delayed-phase MRI demonstrating washout of contrast enhancement.

**Fig. 3:**
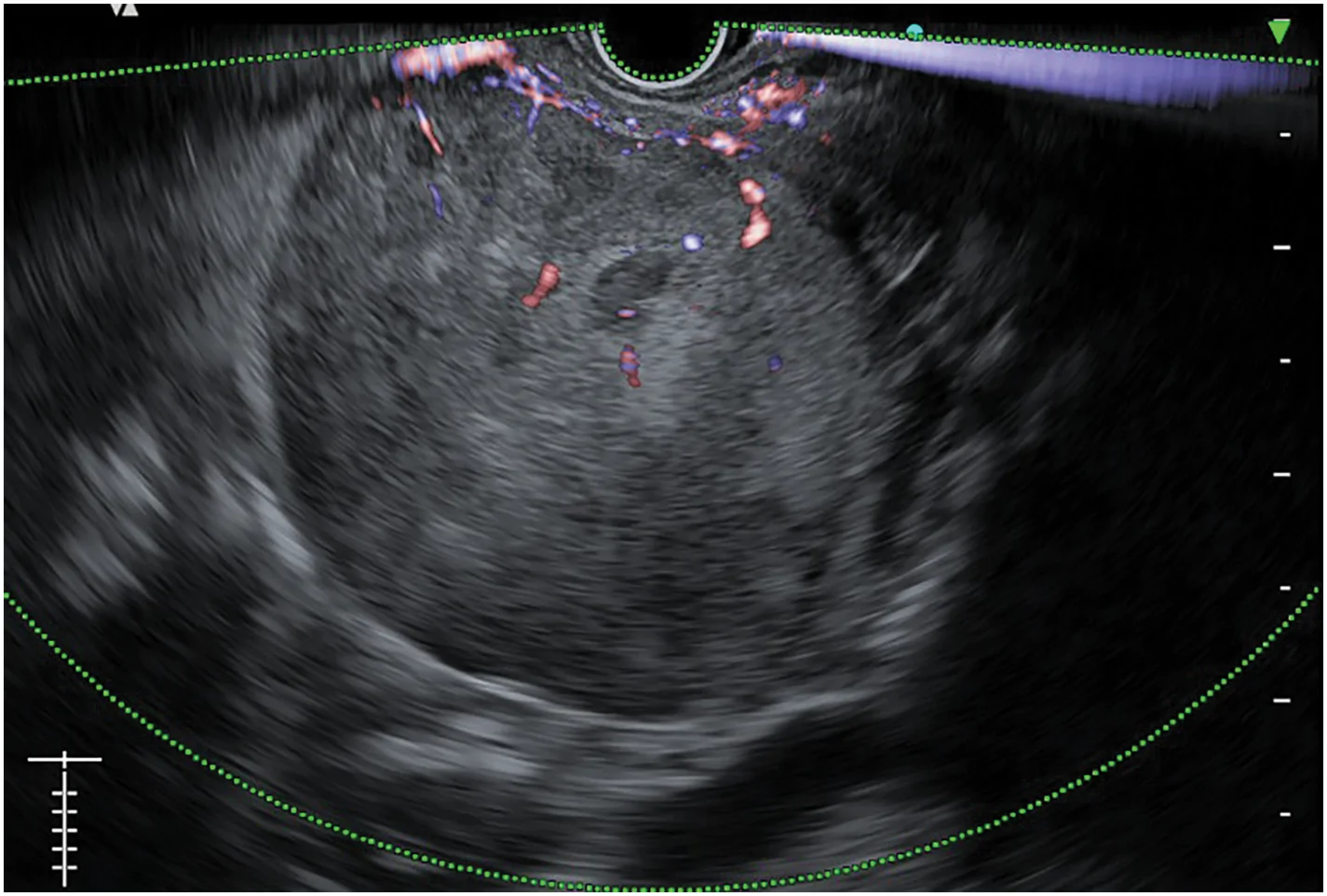
Endoscopic ultrasonography findings. Endoscopic ultrasonography demonstrated a well-defined solid mass in the pancreatic body with predominantly homogeneous echogenicity. Color Doppler imaging revealed intratumoral vascular flow.

**Fig. 4:**
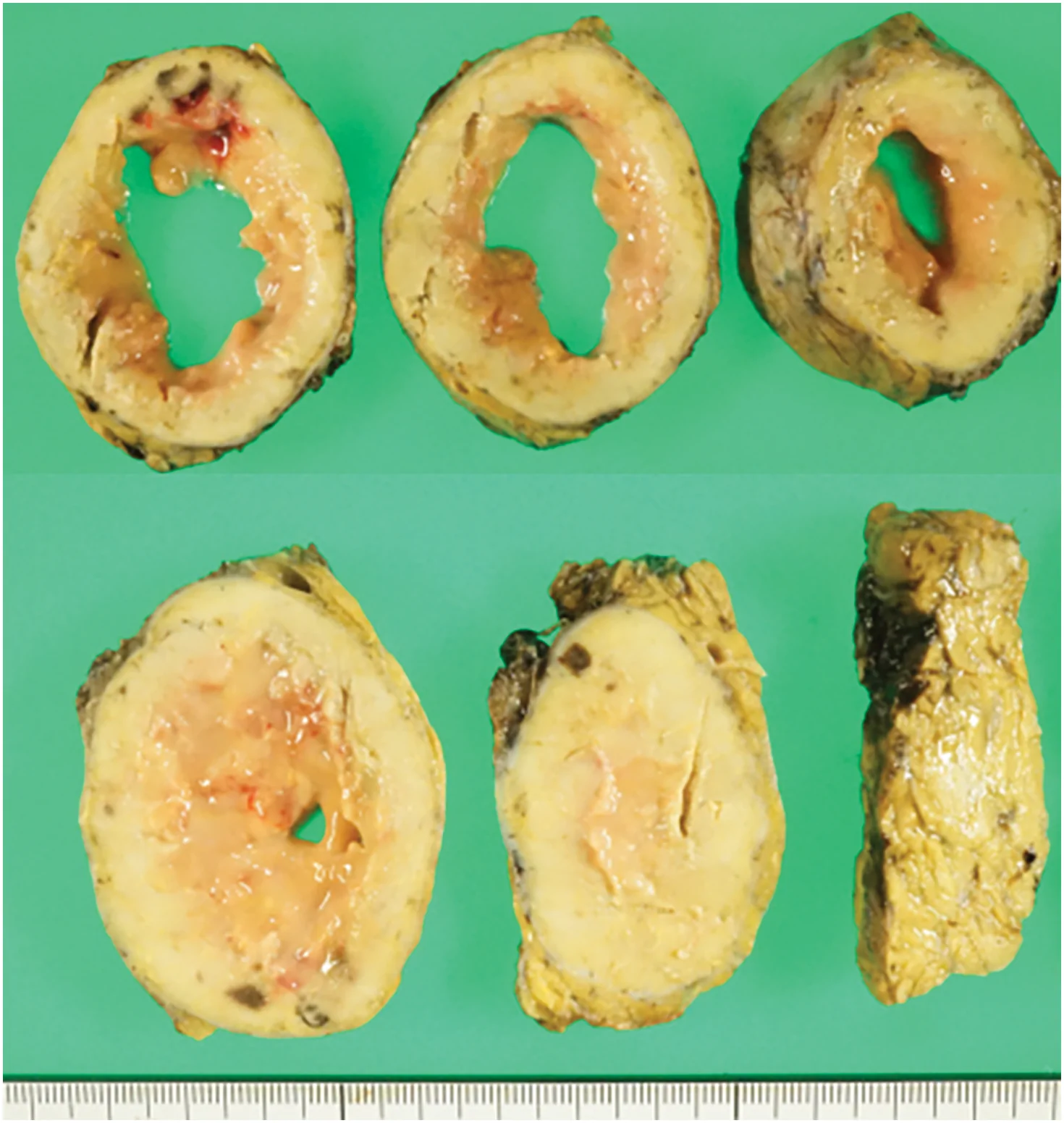
Macroscopic findings of the resected specimen. The cut surface showed a well-circumscribed, yellowish-white solid tumor.

**Fig. 5:**
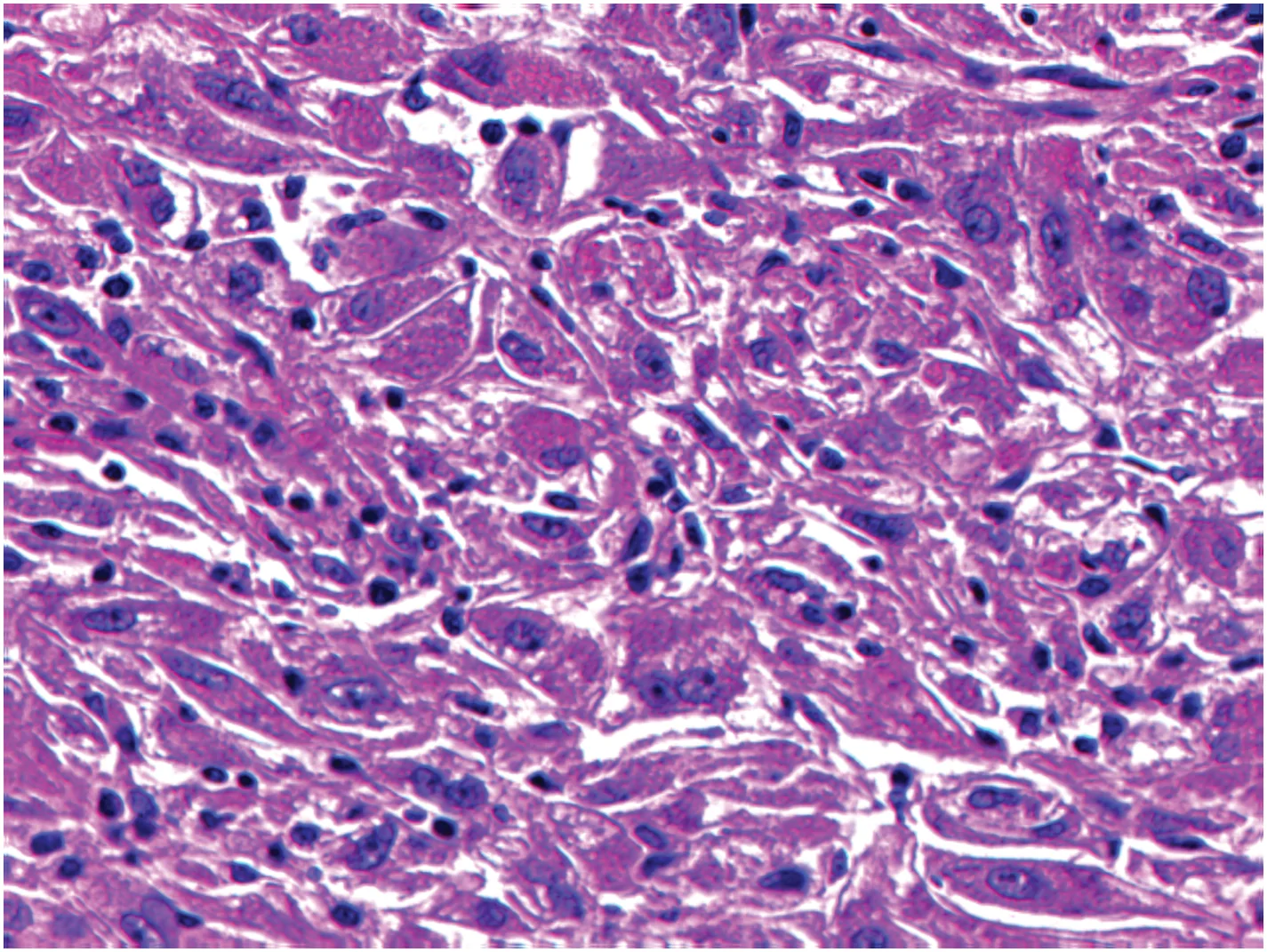
Histopathological findings (hematoxylin and eosin staining). The tumor was composed of spindle-shaped and epithelioid cells with clear to eosinophilic cytoplasm arranged around blood vessels.

**Fig. 6:**
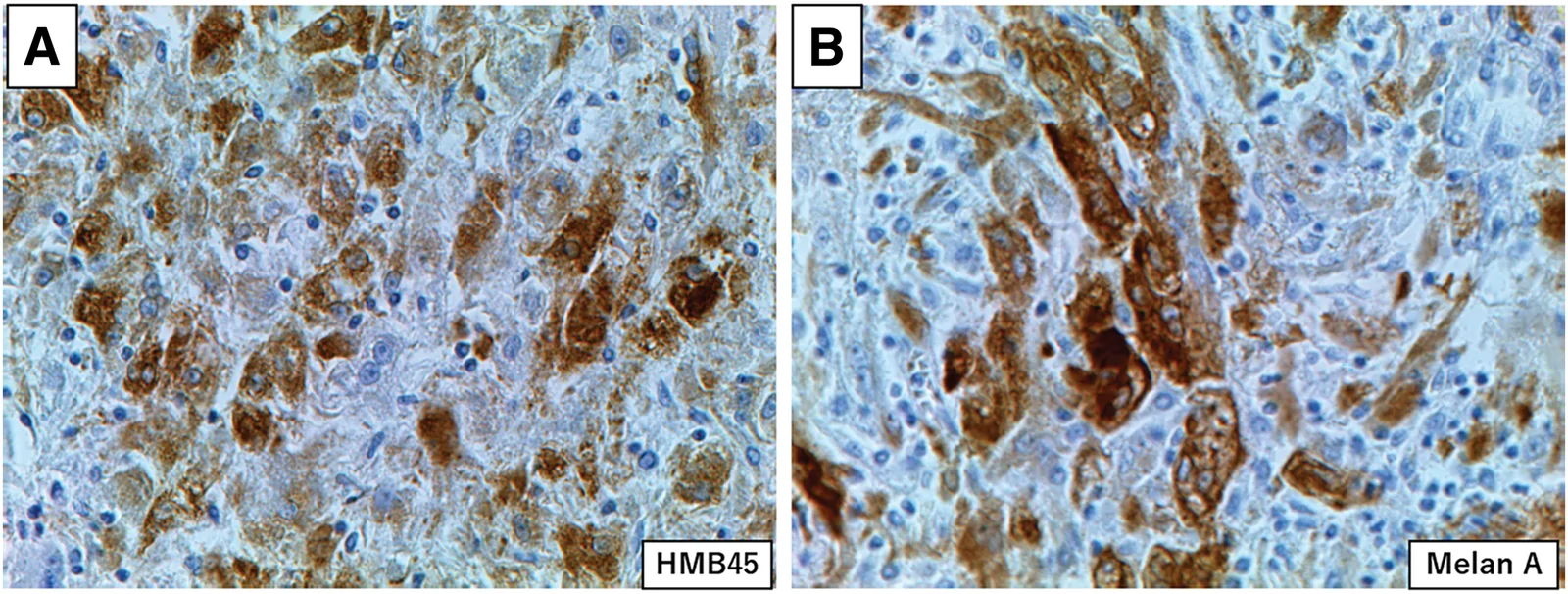
Immunohistochemical findings. (**A**, left panel) Tumor cells showed diffuse positivity for HMB45. (**B**, right panel) Tumor cells were positive for Melan-A. HMB45, human melanoma black 45

## DISCUSSION

PEComas are rare mesenchymal neoplasms characterized by the co-expression of melanocytic and smooth muscle markers.^1)^ Most pancreatic PEComas have been reported as isolated case reports or small case series. The present case and our literature review provide 2 clinically relevant messages. First, pancreatic PEComa should be considered in the differential diagnosis of hypervascular pancreatic tumors with atypical immunophenotypic findings. Second, recognition of this rare entity may facilitate appropriate immunohistochemical evaluation and individualized surgical planning.

The number of pancreatic PEComas included in previous reviews has varied, probably because of differences in the inclusion of clear cell “sugar” tumors and angiomyolipomas in earlier reports, possible duplication of cases, and variability in the pathological information available in the original publications. To better characterize pancreatic PEComa, we searched PubMed and the Japanese medical literature database (Ichushi-Web) through January 2026 using the keywords “pancreas,” “pancreatic,” and “PEComa”.^2–26)^ Original reports describing primary pancreatic PEComas with available clinical information were considered eligible. Conference abstracts without full-text articles and duplicate publications describing the same patient were excluded, leaving 26 individual cases for review.^2–26)^ Clinicopathological characteristics and clinical outcomes were extracted from the original reports, and 22 cases, including the present case, contained sufficient pathological information for reclassification according to the Folpe criteria, allowing a standardized assessment of pathological risk.

The imaging findings of pancreatic PEComa are nonspecific. Pancreatic PEComas are typically described as well-circumscribed hypervascular tumors with arterial-phase enhancement.^4)^ In the present case, contrast-enhanced CT and dynamic MRI demonstrated a well-defined hypervascular tumor in the pancreatic body with relatively decreased central enhancement, which corresponded to focal coagulative necrosis in the resected specimen. However, these findings overlap considerably with those of pancreatic neuroendocrine neoplasms, solid pseudopapillary neoplasms, and acinar cell carcinoma, making a diagnosis based on imaging findings alone difficult. Among the 15 patients who underwent EUS-TA, 11 (73%) were correctly diagnosed with PEComa before surgery (**Table 1**), highlighting the value of tissue acquisition combined with appropriate immunohistochemical evaluation.

**Table 1:** Clinicopathological characteristics and pathological risk factors of reported pancreatic PEComas

No.	Author	Year	Age	Sex	Location	Size (cm)	EUS-TA	Preoperative diagnosis	Surgery	Ki-67 (%)	Necrosis	Mitosis (/50 HPF)	Vascular invasion	Folpe classification (re-evaluated)	Metastasis	Outcome	Follow-up (months)	Reference
1	Hirabayashi	2009	47	F	Head	1.7	No	NET or ACC	PPPD	<1	–	1/10 HPF	–	B	–	NED	12	^5)^
2	Baez	2009	60	F	Body	3.5	Yes	Poorly differentiated carcinoma	DP	N.R.	–	Rare (N.R.)	–	UMP	–	NED	7	^6)^
3	Nagata	2011	52	M	Head	4.0	No	Unknown	PD	N.R.	N.R.	1	–	UMP	Liver	AWD	39	^2)^
4	Zemet	2011	49	M	Head	3.2	Yes	Sugar tumor of the pancreas	PD	<1	–	0	–	B	–	NED	10	^7)^
5	Finzi	2012	62	F	Head	2.5	Yes	Paraganglioma	Total excision	8	N.R.	Rare (N.R.)	–	B	–	NED	5	^8)^
6	Al-Haddad	2013	38	F	Head	1.8	Yes	PEComa	PD	N.R.	N.R.	N.R.	–	N.R.	N.R.	N.R.	N.R.	^9)^
7	Okuwaki	2013	43	F	Body/tail	10.0	Yes	PEComa	DP	<5	+	N.R.	–	M	–	NED	7	^10)^
8	Mourra	2013	51	F	Head	6.0	Yes	PEComa	PD	N.R.	+	2	+	M	Liver	AWD	6	^3)^
9	Yamamoto	2014	44	F	Body	2.4	No	NET	DP	<1	–	Almost absent (N.R.)	N.R.	B	N.R.	NED	N.R.	^11)^
10	Petrides	2015	17	F	Head	4.2	Yes	NET	PPPD	N.R.	–	Infrequent (N.R.)	–	B	–	NED	18	^12)^
11	Mizuuchi	2016	61	F	Head/body	6–7	No	NET/ACC/SPN	PD	<1	–	0	–	UMP	–	NED	144	^13)^
12	Collins	2016	54	F	Head/body	2.6	Yes	PEComa	MP	<1	–	0	–	B	–	NED	27	^14)^
13	Zhang	2017	43	F	Head	11.5	No	NET	PD + partial hepatectomy + SMV replacement	5	+	Easily identified (N.R.)	+	M	–	NED	18	^15)^
14	Zizzo	2017	68	M	Head	2.8	Yes	PEComa	No surgery	<1	–	0	–	B	–	AWD	20	^4)^
15	Takahashi	2018	52	F	Head	3.6	No	NET	SSPPD	≤1	–	<1	–	B	–	NED	31	^16)^
16	Sangiorgio-1	2018	47	F	Body	3.0	Yes	PEComa	No surgery	N.R.	N.R.	N.R.	N.R.	N.R.	N.R.	N.R.	N.R.	^17)^
17	Sangiorgio-2	2018	70	F	Body	2.0	Yes	PEComa	No surgery	N.R.	N.R.	N.R.	N.R.	N.R.	N.R.	N.R.	N.R.	^17)^
18	Uno	2019	49	F	Tail	4.0	Yes	PEComa	DP	<5	–	0	N.R.	B	–	NED	12	^18)^
19	Gondran	2019	17	M	Head	5.5	Yes	PEComa	No surgery	10	N.R.	0	N.R.	M	–	AWD	42	^19)^
20	Ulrich	2020	49	F	Body	2.5	Yes	PEComa	LDP	N.R.	N.R.	N.R.	N.R.	N.R.	N.R.	N.R.	N.R.	^20)^
21	Colón Rodríguez	2020	50	F	Body	1.3	Yes	Atypical clear cells; definitive diagnosis not possible	LDP	<1	–	1/10 HPF	–	B	–	NED	18	^21)^
22	Geng	2021	40	F	Body	2.3	No	SPN	PPPD	5	–	≤1	–	B	–	NED	30	^22)^
23	Harrison	2022	39	M	Tail	3.2	No	N.R.	LDP	N.R.	N.R.	N.R.	N.R.	N.R.	–	NED	N.R.	^23)^
24	Nogueira Sixto	2022	68	M	Head	7.8	No	NET	PPPD	N.R.	–	<1	N.R.	UMP	–	NED	18	^24)^
25	Tsukita	2024	74	M	Tail	3.0	No	Malignant pancreatic tumor	DP	N.R.	–	0	–	B	–	NED	13	^25)^
26	Gilligan	2025	8	F	Head	3.2	Yes	PEComa	PD	N.R.	–	≤1	–	B	–	NED	12	^26)^
27	Present case		72	F	Body	6.3	Yes	ACC	DP	5	+	0	–	M	–	NED	18	

The Folpe classification was re-evaluated by the present authors based on the pathological findings described in the original reports and was applied only to cases with sufficient pathological information.

ACC, acinar cell carcinoma; AWD, alive with disease; B, benign; DP, distal pancreatectomy; EUS-TA, endoscopic US-guided tissue acquisition; F, female; HPF, high-power field; LDP, laparoscopic distal pancreatectomy; M, male/malignant; MP, middle pancreatectomy; NED, no evidence of disease; NET, neuroendocrine tumor; N.R., not reported; PD, pancreatoduodenectomy; PEComas, perivascular epithelioid cell tumors; PPPD, pylorus-preserving pancreatoduodenectomy; SMV, superior mesenteric vein; SPN, solid pseudopapillary neoplasm; SSPPD, subtotal stomach-preserving pancreatoduodenectomy; UMP, uncertain malignant potential

The diagnosis of PEComa requires demonstration of melanocytic and myogenic differentiation, typically by positivity for HMB45, Melan-A, and α-SMA together with negativity for epithelial and neuroendocrine markers.^1)^ In the present case, weak trypsin positivity in the preoperative EUS-TA specimen initially suggested acinar cell carcinoma. Although the weak staining was not typical of acinar cell carcinoma, no other specific differential diagnosis was considered at that time. However, the absence of epithelial and neuroendocrine marker expression was also atypical for acinar cell carcinoma. Trypsin expression has not been established as a characteristic immunophenotypic feature of PEComa,^1)^ and a previously reported pancreatic PEComa examined for acinar markers was negative for trypsin.^13)^ Therefore, the reason for the weak trypsin positivity in the present case remains unclear. Weak or focal trypsin immunoreactivity should be interpreted cautiously in small cytological or biopsy specimens. Normal pancreatic acinar cells strongly express trypsin, and trypsin immunohistochemistry may exhibit substantial background staining. Thus, entrapped or contaminating nonneoplastic acinar cells or nonspecific background staining may be misinterpreted as weak tumor-cell positivity in limited specimens. False-positive cytological diagnoses of acinar cell carcinoma due to the misinterpretation of nonneoplastic acinar cells or isolated trypsin positivity have also been reported. ^27, 28)^ Accordingly, weak trypsin positivity alone, in the absence of characteristic acinar morphology, should not be considered sufficient evidence of acinar differentiation, and confirmation with additional acinar markers, such as BCL10 and chymotrypsin, is warranted. Examination of the resected specimen in the present case demonstrated diffuse positivity for HMB45 and Melan-A, weak positivity for α-SMA, and negativity for S-100, establishing the diagnosis of pancreatic PEComa. Because melanocytic markers are not routinely included in the initial immunohistochemical panel for hypervascular pancreatic tumors, additional staining for HMB45 and Melan-A should be considered when the morphological and immunohistochemical findings are inconsistent with the suspected diagnosis. Our case and literature review suggest that recognizing such atypical findings and applying an expanded immunohistochemical panel may facilitate the diagnosis of pancreatic PEComa and help avoid its misclassification as another hypervascular pancreatic neoplasm.

Correct preoperative diagnosis may influence the operative strategy for pancreatic PEComa. In the present case, distal pancreatectomy with regional lymph node dissection was performed because acinar cell carcinoma was strongly suspected preoperatively. However, lymph node metastasis appears to be uncommon in pancreatic PEComa, and the indications for lymph node dissection have not been established. Complete surgical resection remains the treatment of choice for PEComa; however, the optimal extent of resection has not been established because of its rarity. Compared with pancreatic PEComa, uterine PEComa has been more extensively investigated because of the larger number of reported cases. In uterine PEComa, individualized surgical strategies, including organ-preserving mass resection, have been proposed in selected patients, particularly when fertility preservation is desired.^29)^ Although the clinical setting differs from that of pancreatic PEComa, these reports suggest that surgical management should be tailored according to tumor characteristics and the clinical context. Therefore, establishing the correct diagnosis before surgery may help determine the appropriate extent of pancreatic resection and lymph node dissection. In the present case, given the tumor size of 63 mm and its location in the pancreatic body, parenchyma-sparing surgery would not have been considered appropriate, and distal pancreatectomy would likely have been required irrespective of the preoperative diagnosis. Nevertheless, preoperative identification of pancreatic PEComa may allow consideration of parenchyma-sparing procedures in selected patients with small, localized tumors. Indeed, central pancreatectomy has been successfully performed for a small pancreatic PEComa after the correct diagnosis had been established preoperatively, suggesting that parenchyma-sparing pancreatectomy may be feasible in carefully selected patients.

Although most reported pancreatic PEComas have followed a favorable clinical course, metastatic disease has been reported,^2, 3)^ indicating that pancreatic PEComa should not be regarded as an invariably benign neoplasm. The Folpe classification, originally developed for gynecologic and soft tissue PEComas, is currently the most widely used pathological risk classification for estimating malignant potential.^30)^ To better characterize the pathological features of pancreatic PEComa, we re-evaluated 22 cases, including the present case, according to the Folpe criteria. Four tumors were classified as malignant and 4 as having UMP. One patient in each category subsequently developed liver metastasis, whereas the remaining patients remained recurrence-free during the reported follow-up. Notably, both patients with metastatic disease were alive 6 and 39 months after recurrence, respectively. However, because only 2 cases with metastatic disease were identified, comparisons of individual pathological features between metastatic and nonmetastatic tumors are necessarily descriptive and hypothesis-generating. Accordingly, the prognostic significance of coagulative necrosis and mitotic activity and the utility of the Folpe classification cannot be determined from the available data. These observations suggest that the clinical course of pancreatic PEComa is heterogeneous but should be interpreted cautiously. Additional well-documented cases with long-term follow-up are required to clarify the relationships among individual pathological features, the Folpe classification, and clinical outcomes.

## CONCLUSIONS

Primary pancreatic PEComa is a rare neoplasm that should be considered in the differential diagnosis of hypervascular pancreatic tumors with atypical immunophenotypic findings. Appropriate immunohistochemical evaluation, including staining for melanocytic markers, may improve preoperative diagnostic accuracy and facilitate individualized surgical planning. Additional well-documented cases with long-term follow-up are required to clarify the relationships among individual pathological features, the Folpe classification, and clinical outcomes in pancreatic PEComa.

## DECLARATIONS

### Availability of data and material

The dataset supporting the conclusion of this article is included within the article.
